# Giant Coronary Artery Aneurysm Causing Acute Anterior Myocardial Infarction

**DOI:** 10.1155/2016/5180472

**Published:** 2016-11-06

**Authors:** Ahmet Yanık, Uğur Arslan, Murat Akçay, Serdar Menekşe, Uğur Gökmen Yazgan

**Affiliations:** ^1^Department of Cardiology, Samsun Education and Research Hospital, Samsun, Turkey; ^2^Department of Cardiovascular Surgery, Samsun Education and Research Hospital, Samsun, Turkey

## Abstract

A 70-year-old man with hypertension was admitted to our coronary ICU with acute anterior MI. Emergent primary PCI was planned and coronary angiography was performed. LAD artery was totally occluded in the proximal segment just after a huge 32 × 26 mm sized aneurysm. Emergent CABG operation was performed in 75 minutes because of multivessel disease including the RCA and left circumflex artery. Aneurysm was ligated and coronary bypass was performed using LIMA and saphenous grafts. The postoperative course of the patient was uneventful. He was discharged with medical therapy including ASA, clopidogrel, and atorvastatin. He was asymptomatic at his polyclinic visit in the first month.

## 1. Introduction

Coronary artery aneurysm (CAA) is defined as focal or diffuse dilatation of a coronary artery more than 1.5 times of its original diameter [1]. Atherosclerosis is the most common reason of CAA while infection, trauma, vasculitis, Kawasaki's disease, cocaine use, and iatrogenic causes (post-PCI) form the other etiologies [2, 3]. CAAs are usually encountered incidentally during coronary angiography and usually do not cause ischemia unless flow-limiting stenosis is present.

In this report, we present a patient who was admitted to our coronary care unit with acute anterior myocardial infarction (MI). A huge aneurysm was found in left anterior descending (LAD) artery limiting distal blood flow.

## 2. Case

**Figure 1:**
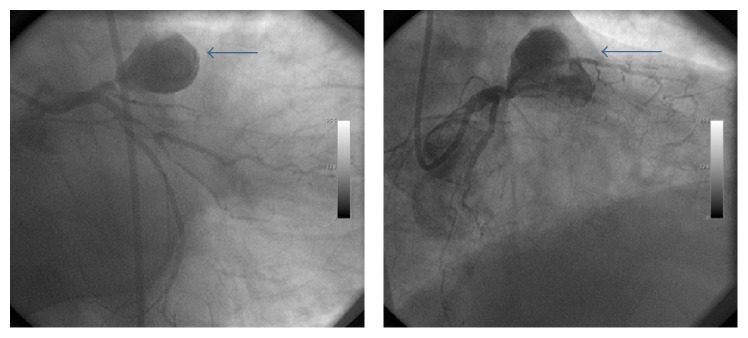
Huge coronary artery aneurysm (32 × 26 mm) in the left anterior descending (LAD) artery. Please note that no flow is present distal to the aneurysm.

A 70-year-old hypertensive man with severe chest pain was admitted to our coronary ICU with acute anterior MI. His vital findings were stable with no prominent finding in his physical examination. Emergent coronary angiography was performed for primary percutaneous intervention of the culprit artery. However, LAD artery was totally occluded in the proximal segment just after a huge 32 × 26 mm sized aneurysm (Figure 1). Then, emergent CABG operation was planned and performed in 75 minutes because of multivessel disease including the RCA and left circumflex artery. Aneurysm was ligated and coronary bypass was performed using LIMA and saphenous grafts. The postoperative course of the patient was uneventful. He was discharged with medical therapy including ASA, clopidogrel, and atorvastatin. He was asymptomatic at his polyclinic visit in the third month.

## 3. Discussion

Coronary artery aneurysms are defined as dilatation of coronary artery diameter ≥1.5 times of its original size and reported to have an incidence of 1.5–4.9% in angiographic series [4]. However, huge coronary aneurysms are rarely reported. In this case, we report a patient with a huge coronary aneurysm which had a diameter approximately 8–10 times greater than the original size of LAD artery. This patient was admitted with acute anterior myocardial infarction and treated with emergent operation.

CAAs are usually asymptomatic and diagnosed incidentally in coronary angiograms [5]. They are mostly located in right coronary artery (RCA) followed by left main, left anterior descending, and circumflex arteries consecutively [5]. Atherosclerosis is the most common cause of CAAs and other etiologies of CAAs are congenital, systemic lupus erythematosus, trauma, mycotic embolism, cocaine use, complicated percutaneous coronary interventions, Kawasaki's disease, and Marfan syndrome [2, 3]. In our case, we thought atherosclerosis is the cause of huge CAA because the patient was old and had atherosclerosis in other coronary arteries.

The prognosis of CAAs differs according to the severity of obstructive coronary artery disease. In asymptomatic patients without severe CAD, conservative approach is recommended. Covered stents may be concerned in eligible symptomatic patients. We did not perform this percutaneous intervention because the aneurysm contained the ostium of the first diagonal artery and the patient had multivessel disease. Surgical approach can be thought in large CAAs with risk of rupture, in CAAs fistulising into a cardiac chamber, or in CAAs accompanying severe coronary artery disease [3]. In our case, the patients had acute anterior myocardial infarction and severe 3-vessel coronary artery disease. Emergent surgery was performed successfully.

In conclusion, huge CAA is a very rare condition and treatment of this aneurysm depends on the clinical findings, location, and size of the CAA. We present this case due to its rarity, interesting clinical admission as acute myocardial infarction, and successful treatment with coronary bypass surgery.
